# Supplementary material for: Distinct genomic and epigenomic features demarcate hypomethylated blocks in colon cancer
Source: BMC Cancer. 2016 Feb 11;16:88. doi: 10.1186/s12885-016-2128-1 (PMC4750190; doi:10.1186/s12885-016-2128-1)

Supplementary Figure 1: Pattern of histone marks near HMB boundaries, (a) H3K4me1

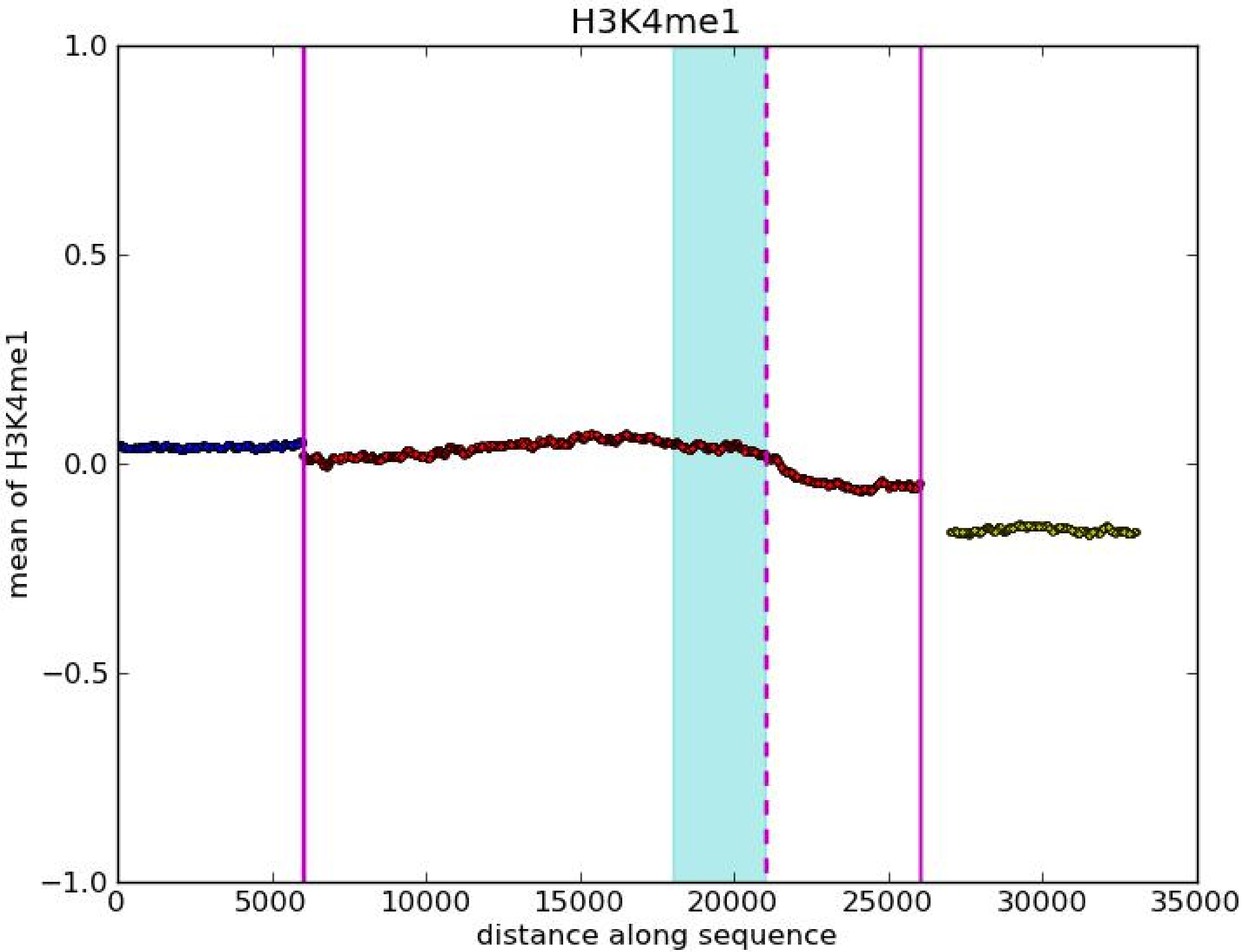

Supplementary Figure 1: Pattern of histone marks near HMB boundaries, (b) H3K9me3

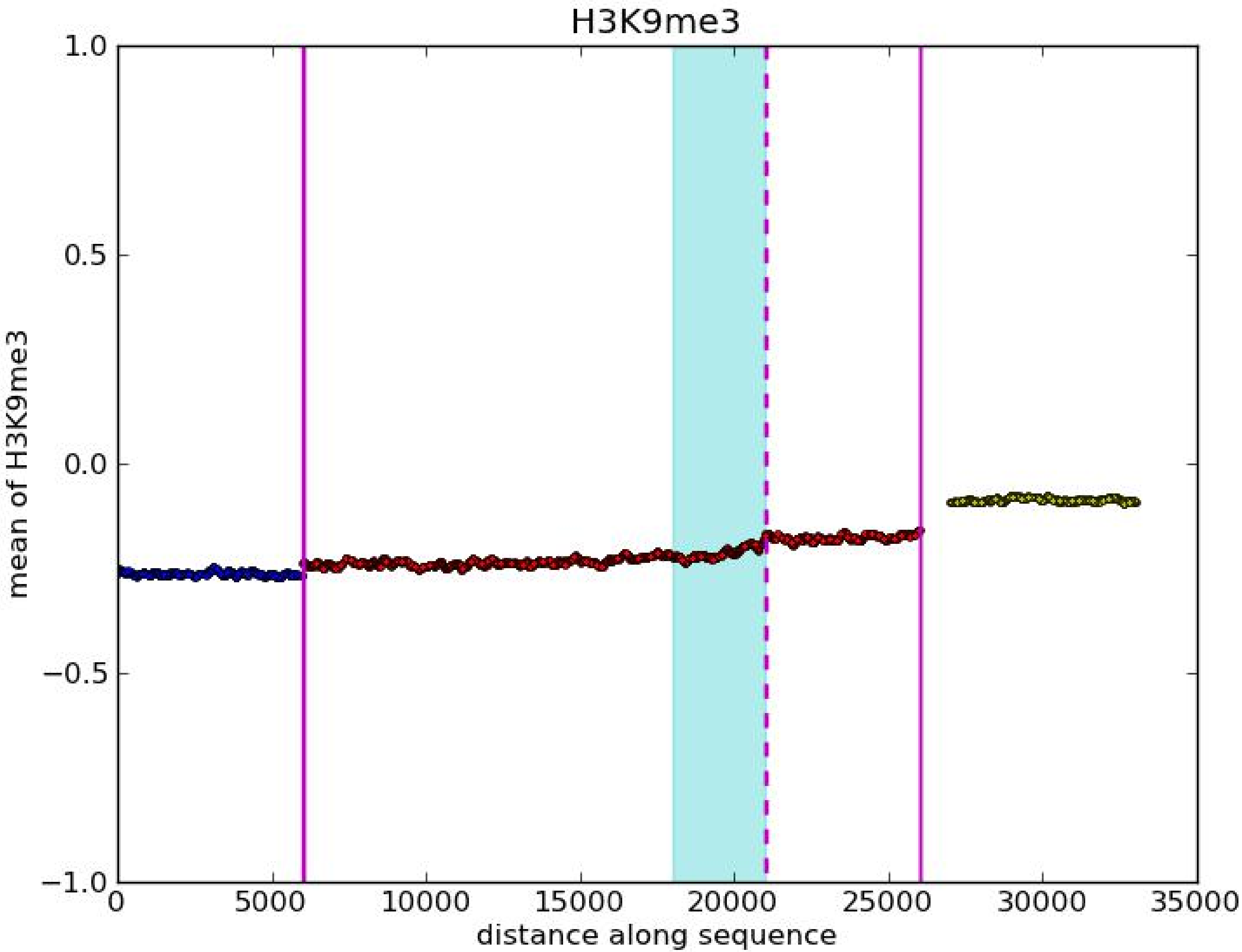

Supplementary Figure 1(c)

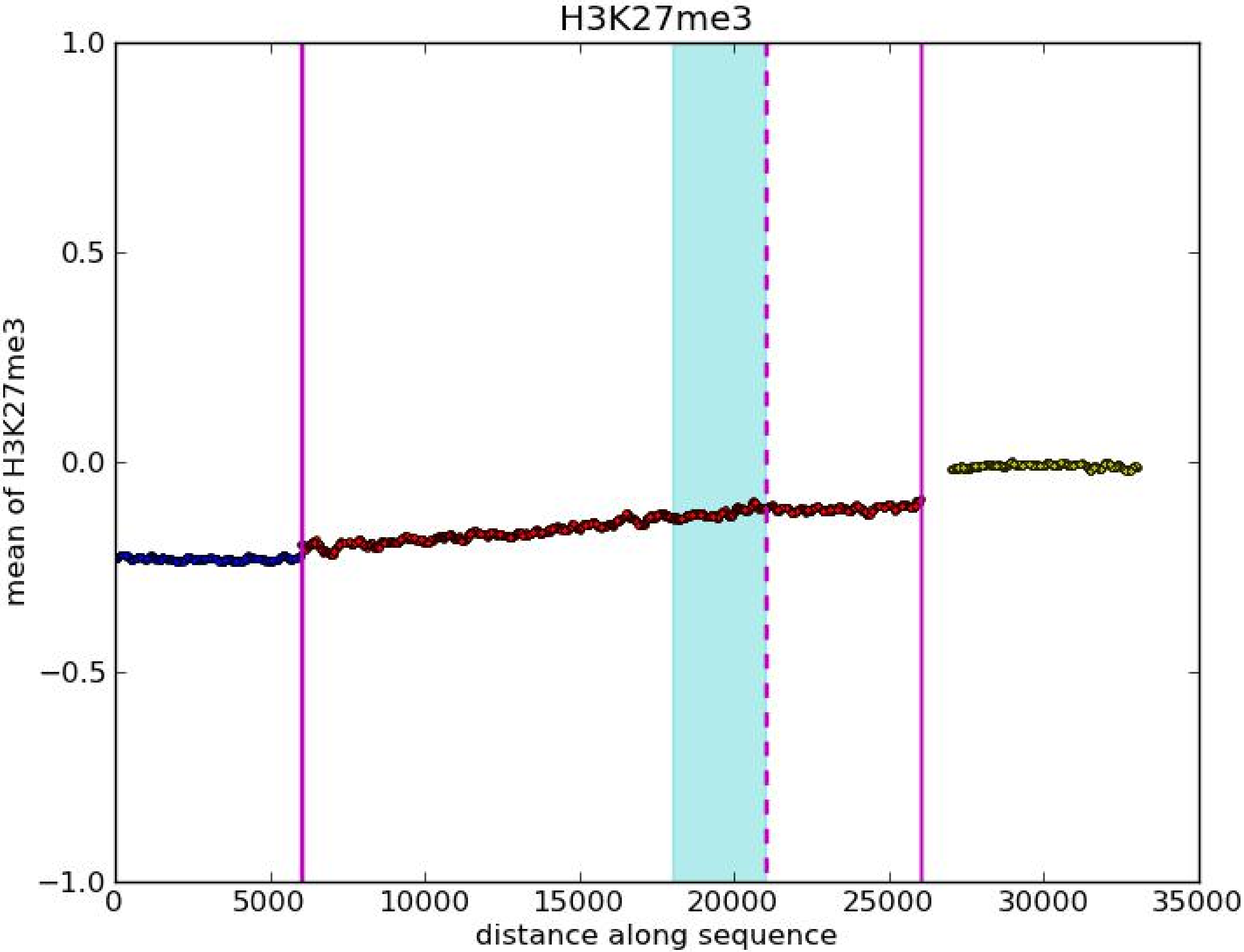

Supplementary Figure 1(d)

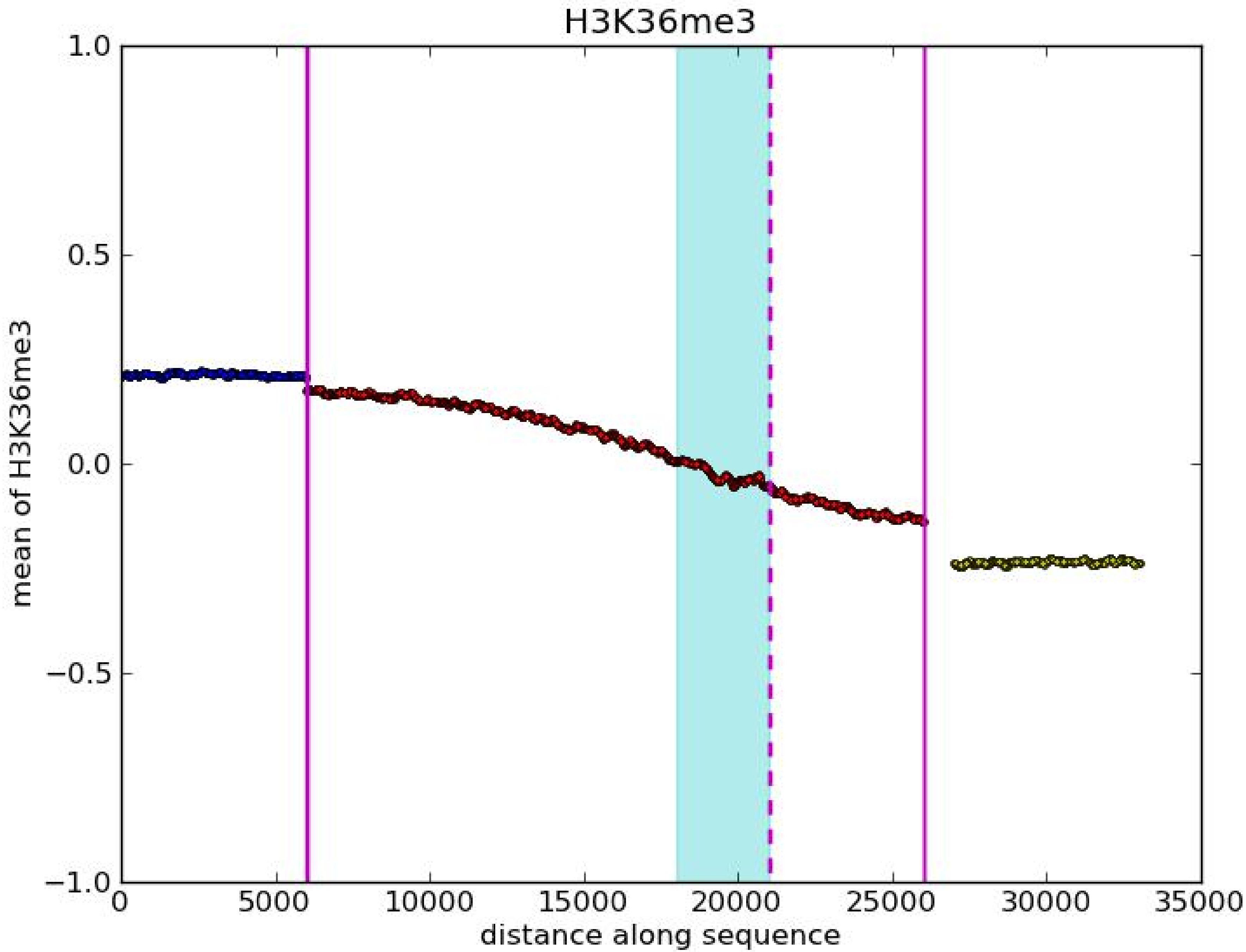

Supplementary Figure 2(a): ROC for HMB boundaries versus inside for the test set.

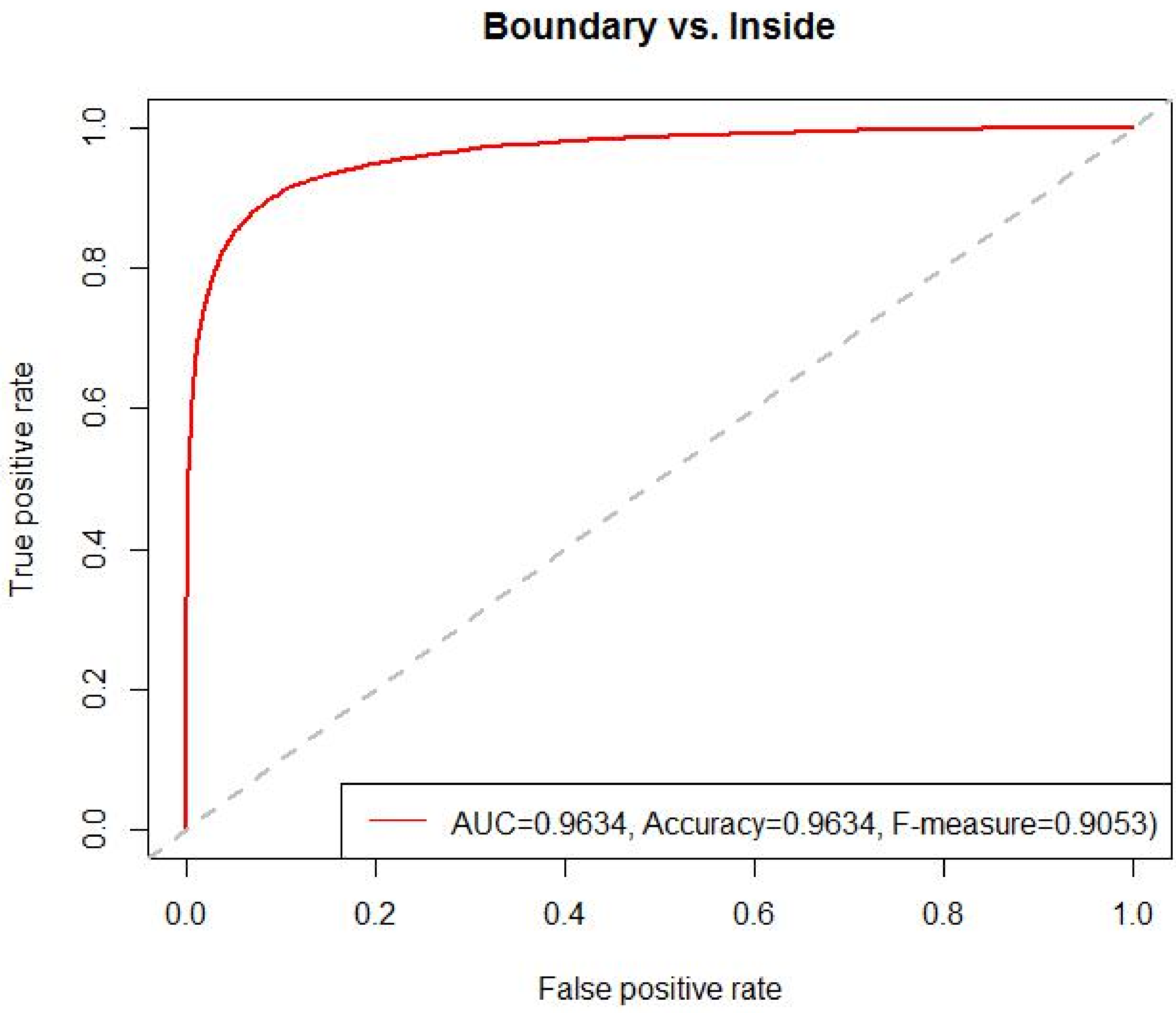

Supplementary Figure 2(b): ROC for HMB boundaries versus outside for the test set.

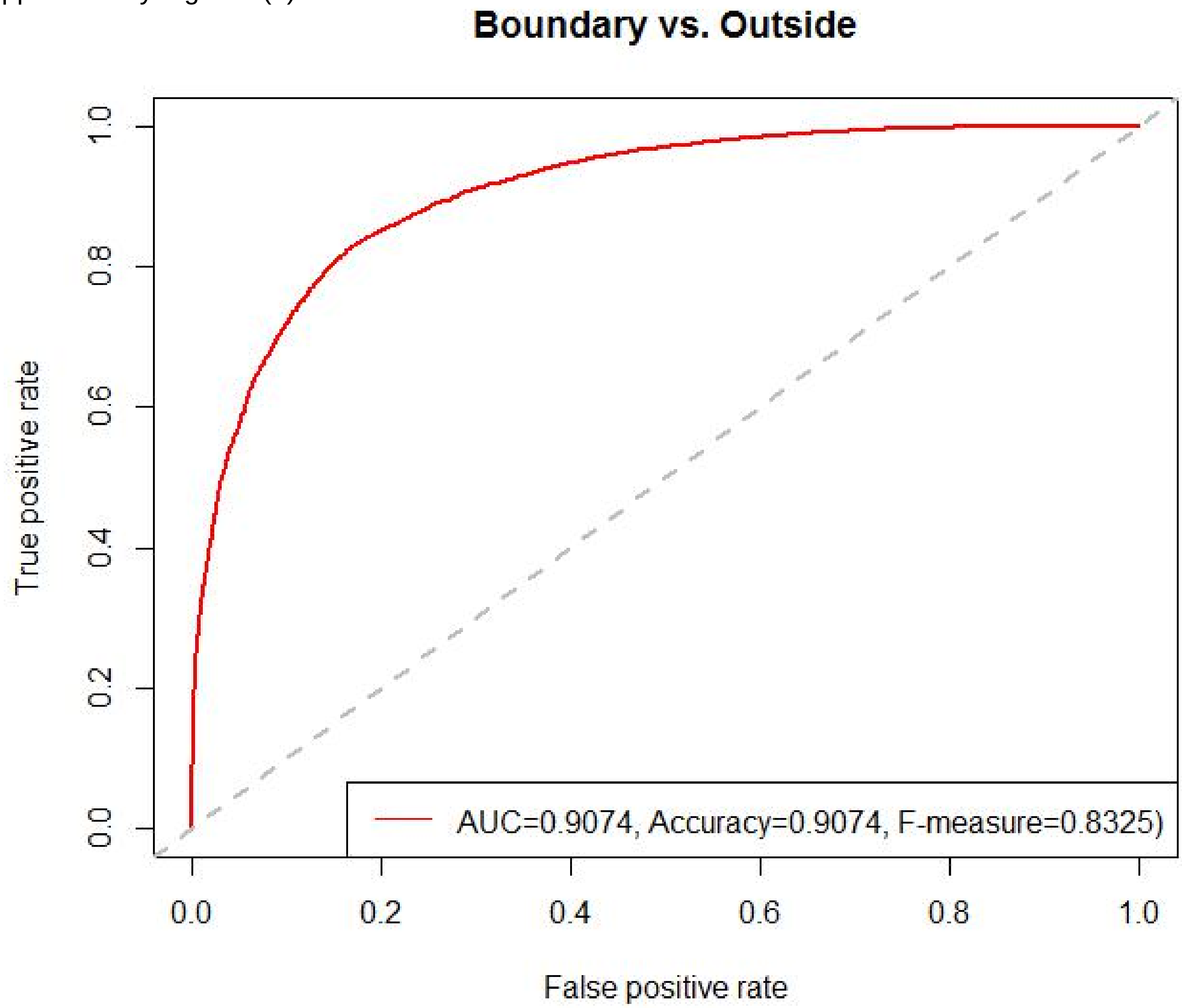

Supplementary Figure 3: Positional profile or Frequency plots for the TF motifs listed in Supplementary Table 4.

boundary: 1.77, promoter: 5.93

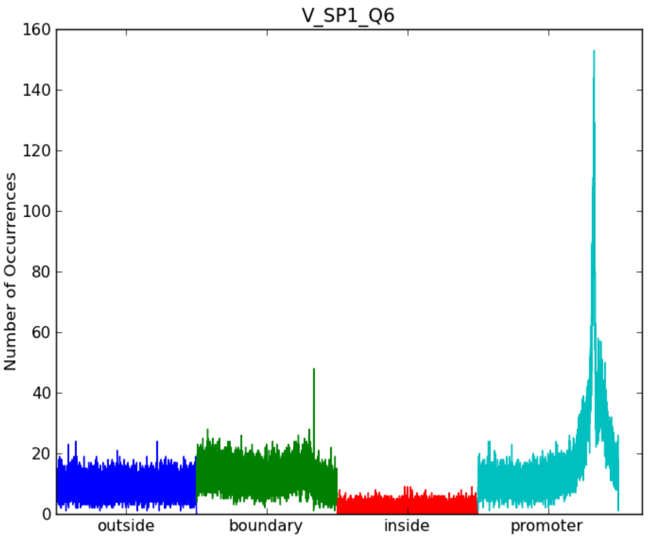

boundary: 2.21, promoter: 3.21

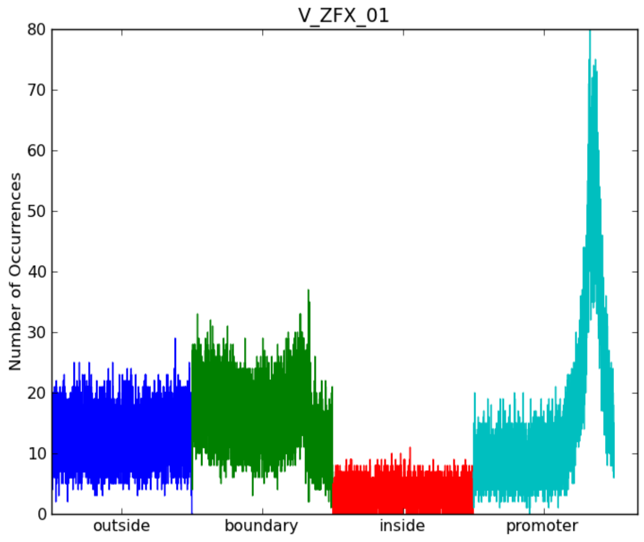

boundary: 2.93, promoter: 1.23

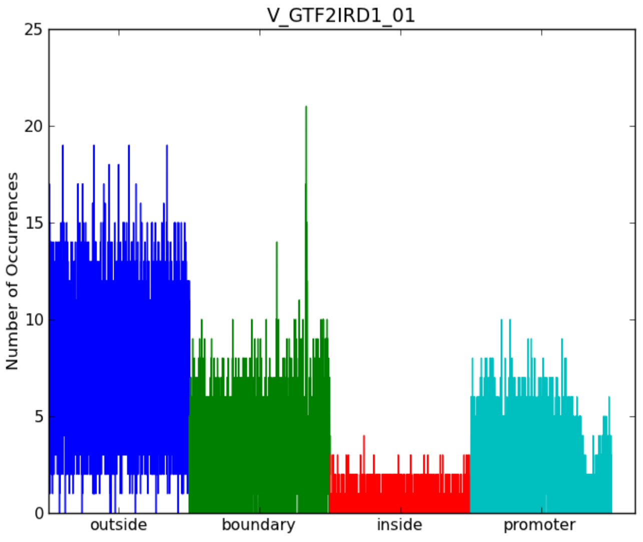

boundary: 3.34, promoter: 1.40

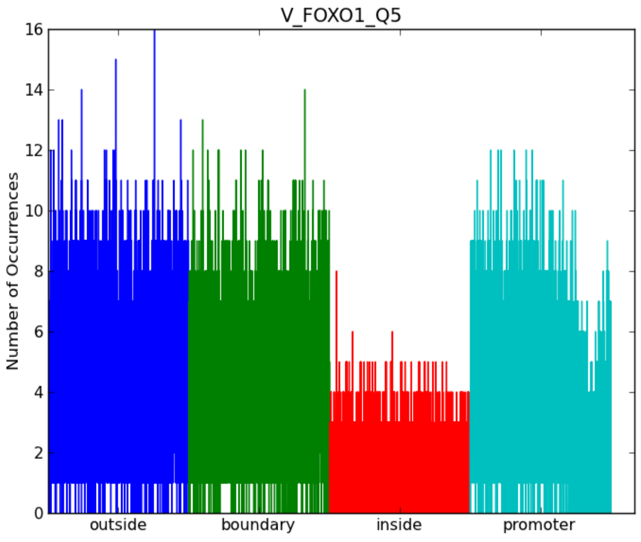

boundary: 3.00, promoter: 4.15

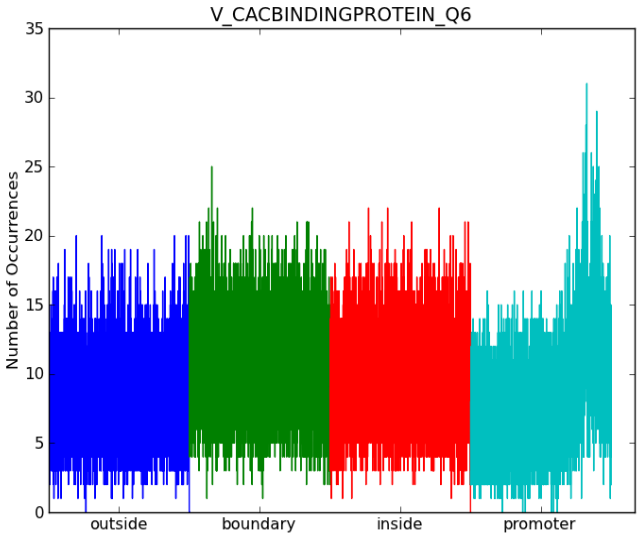

boundary: 1.84, promoter: 4.66

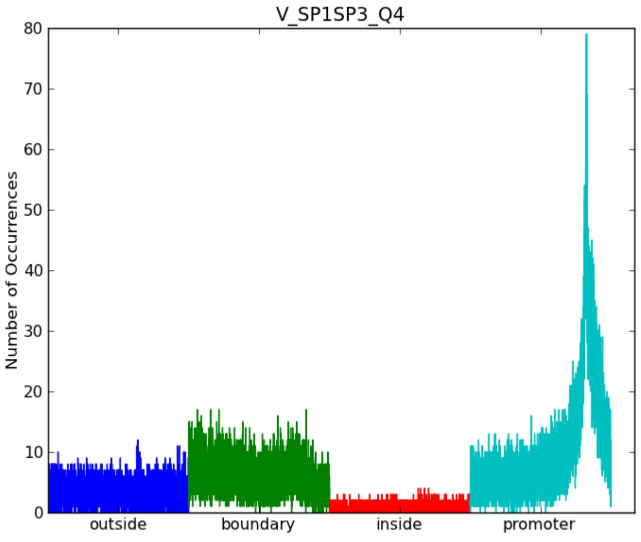

boundary: 1.75, promoter: 5.97

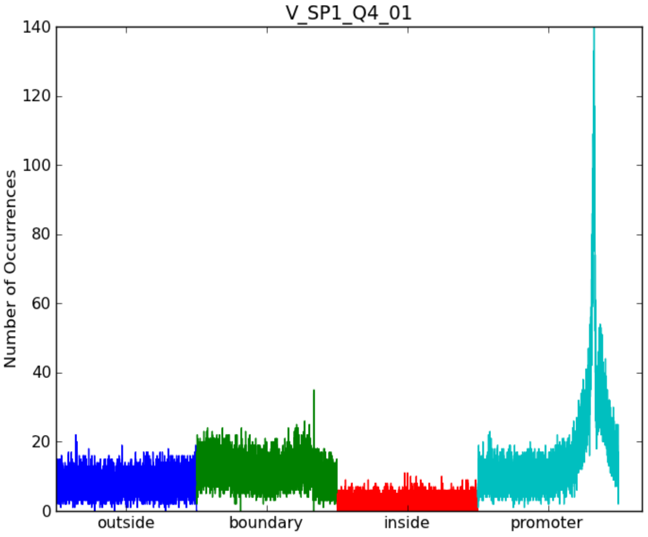

boundary: 4.35, promoter: 1.34

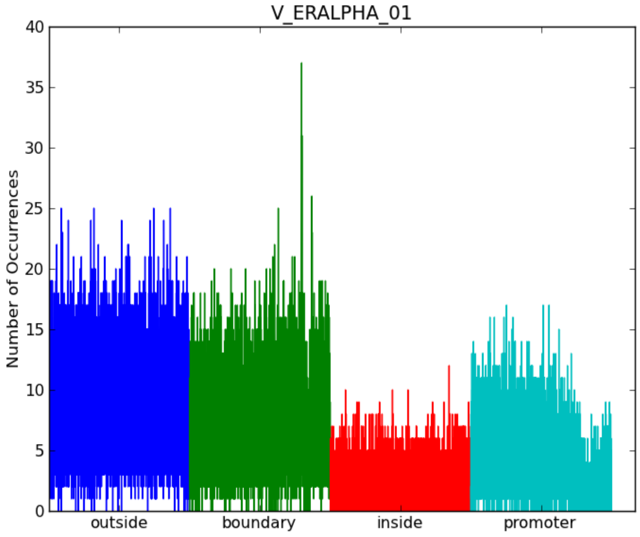

boundary: 2.45, promoter: 5.46

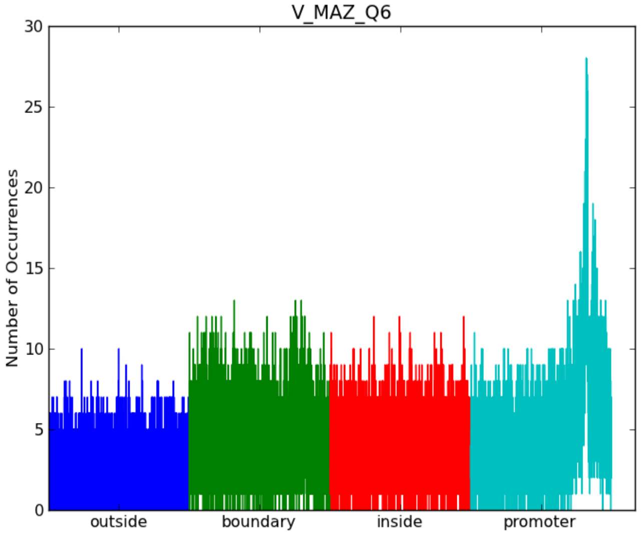

boundary: 3.55, promoter: 3.58

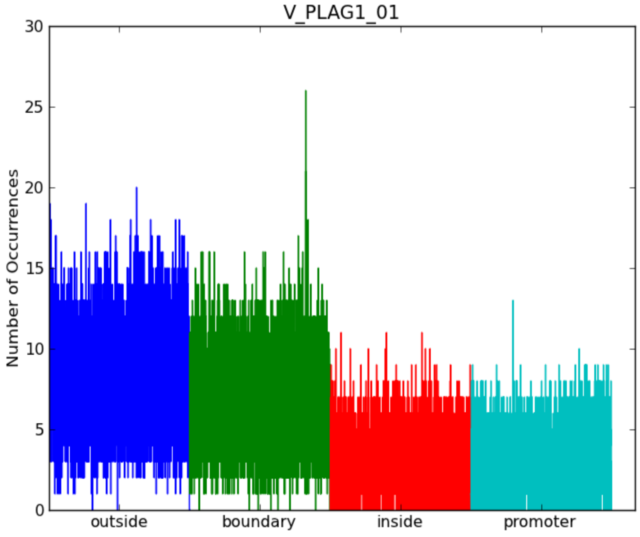

boundary: 2.09, promoter: 6.26

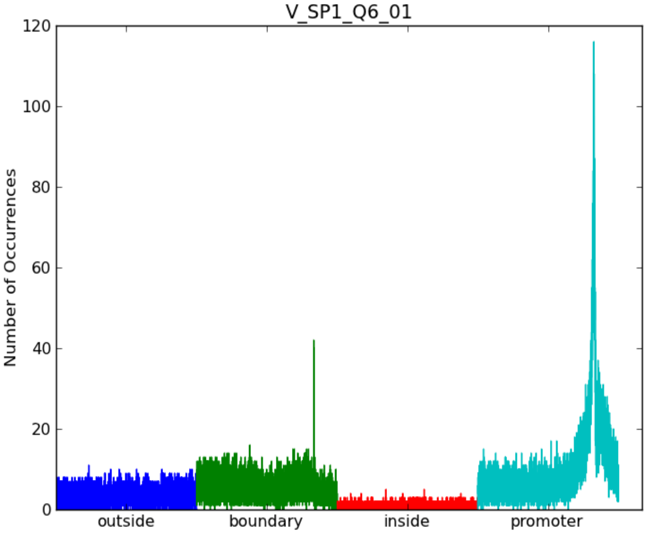

boundary: 2.48, promoter: 6.07

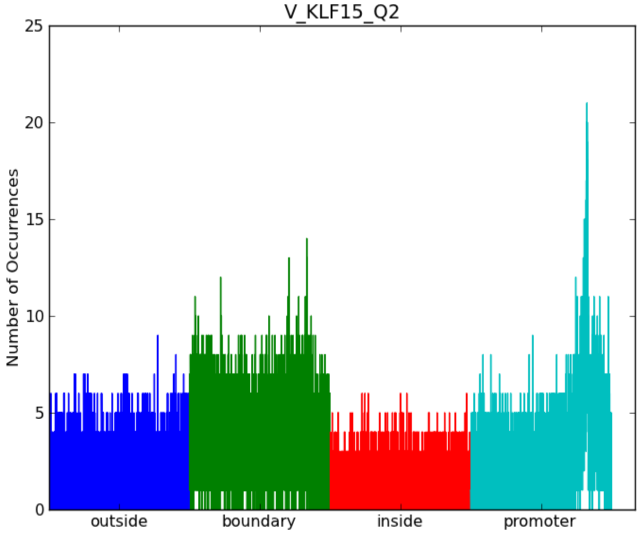

boundary: 1.75, promoter: 5.84

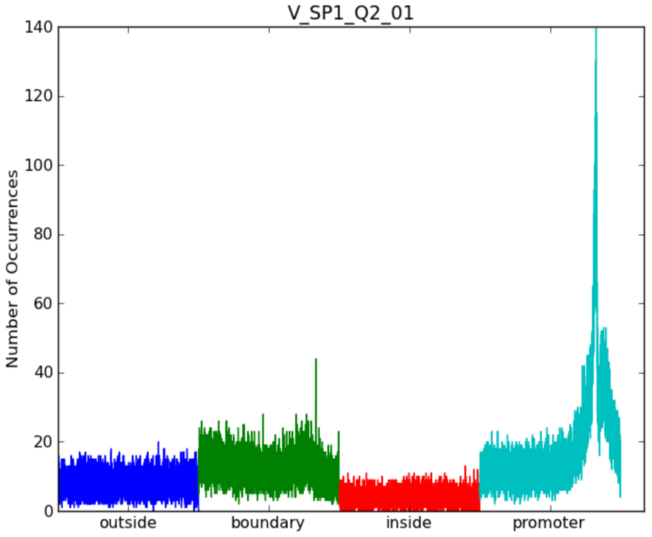

boundary: 2.20, promoter: 5.88

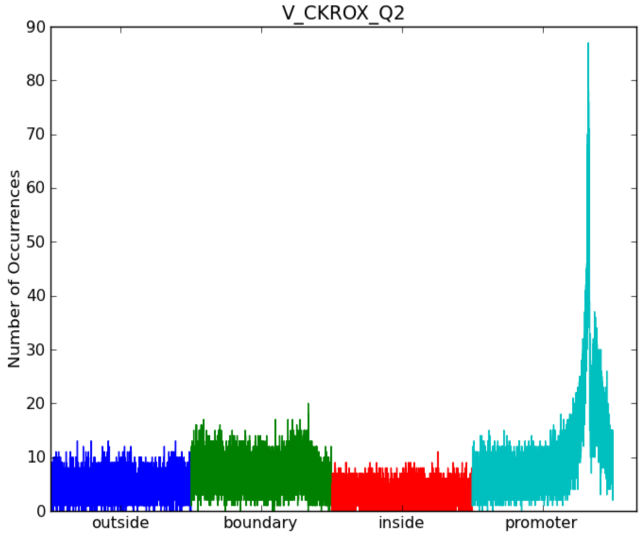

boundary: 1.63, promoter: 5.71

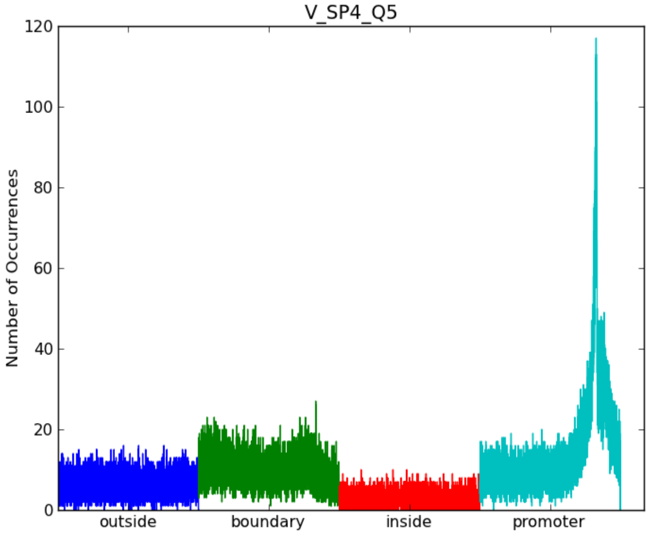

boundary: 2.76, promoter: 4.31

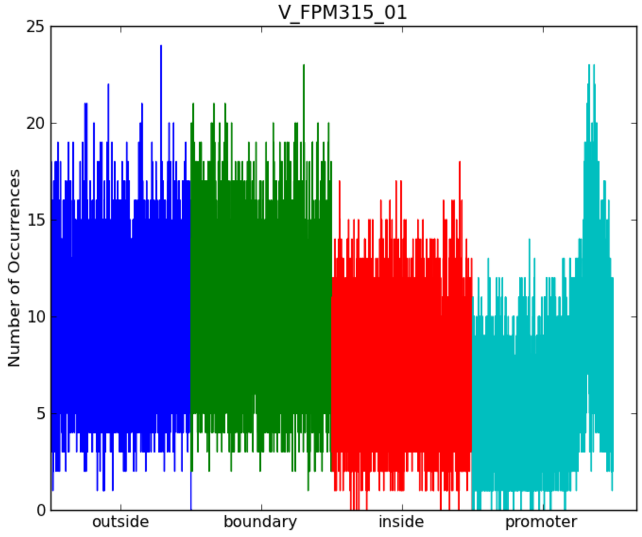

boundary: 2.44, promoter: 5.14

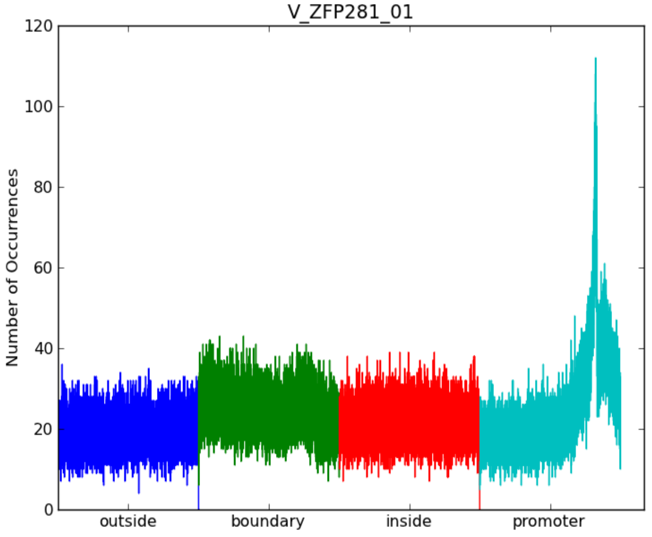

boundary: 2.27, promoter: 6.12

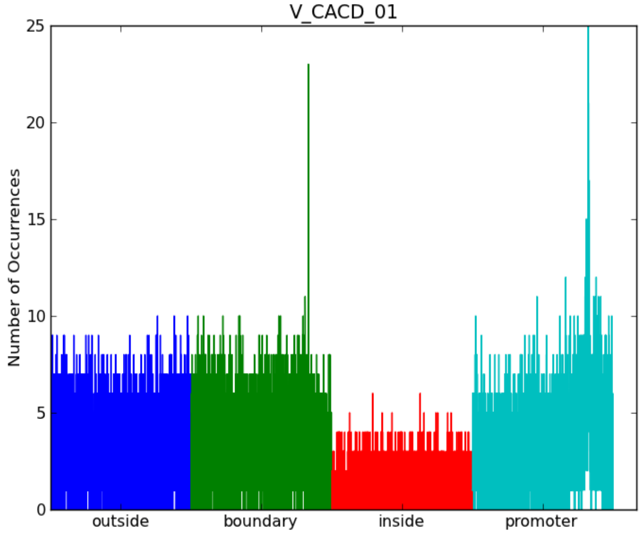

boundary: 2.56, promoter: 6.94

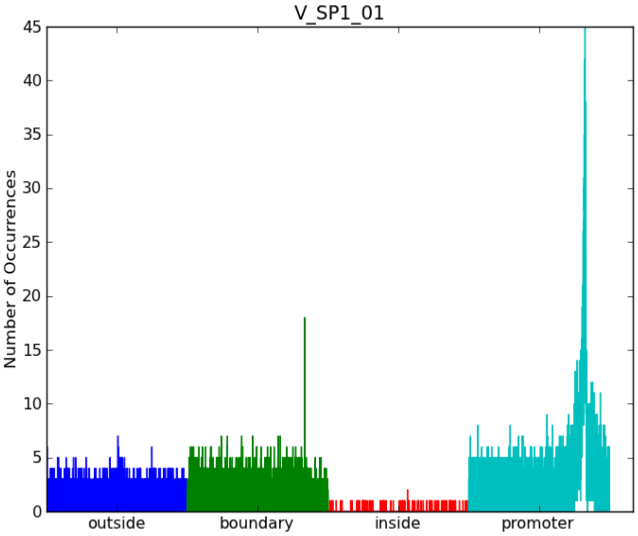

boundary: 1.96, promoter: 4.98

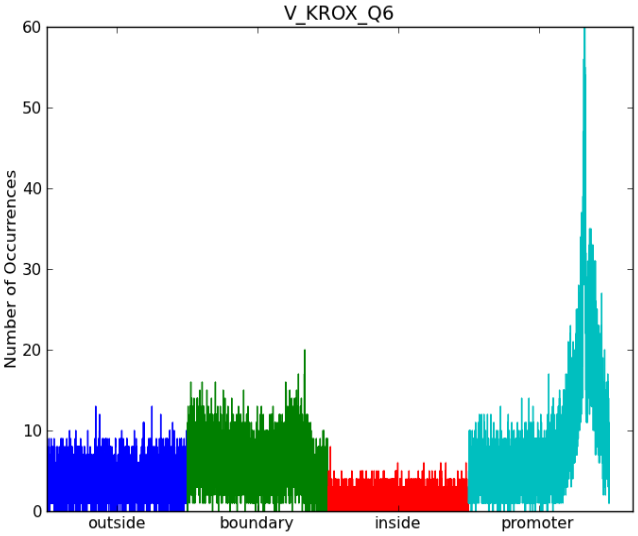

boundary: 5.04, promoter: 1.15

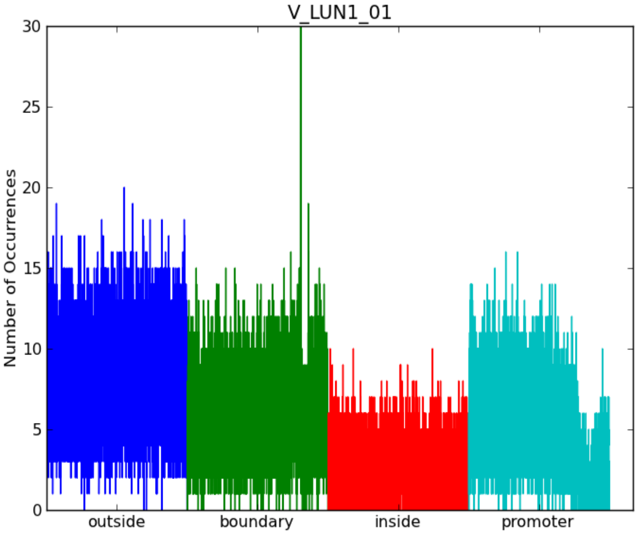

boundary: 2.12, promoter: 5.20

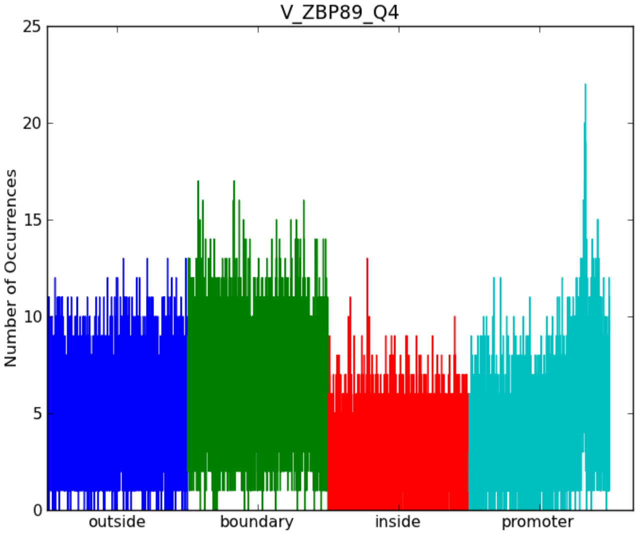

boundary: 3.14, promoter: 1.15

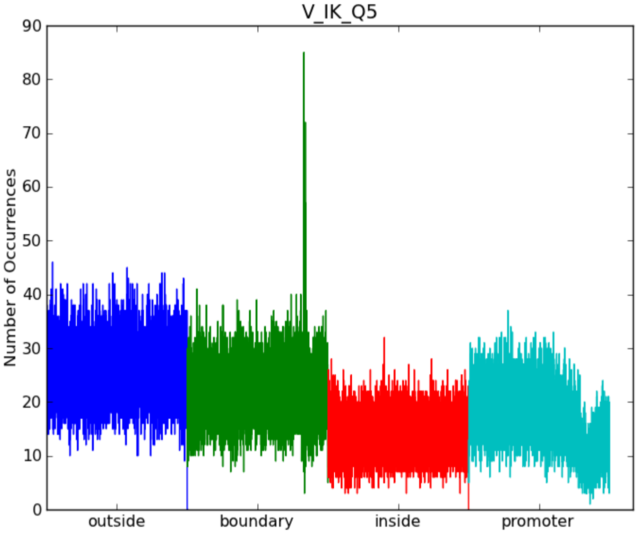

boundary: 2.03, promoter: 3.01

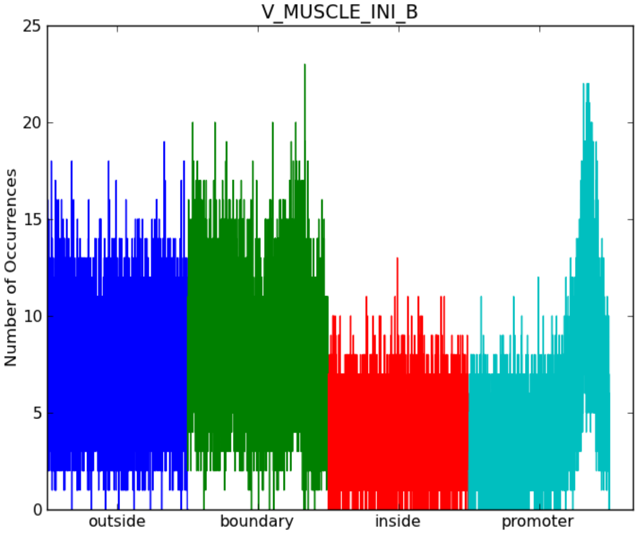

boundary: 1.87, promoter: 5.40

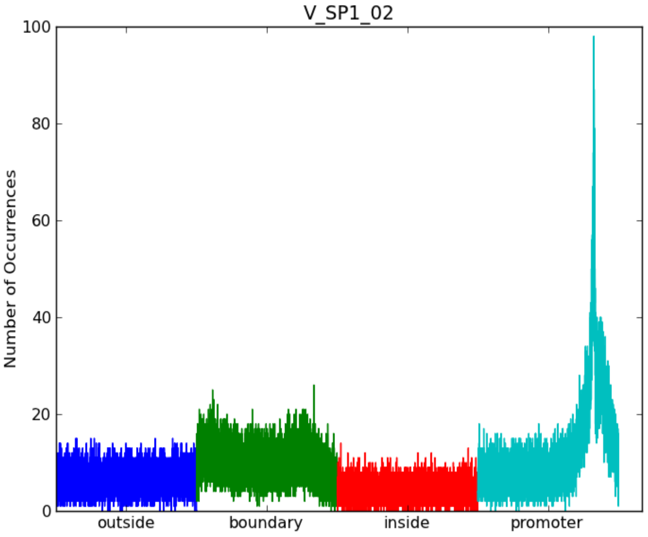

boundary: 2.01, promoter: 4.88

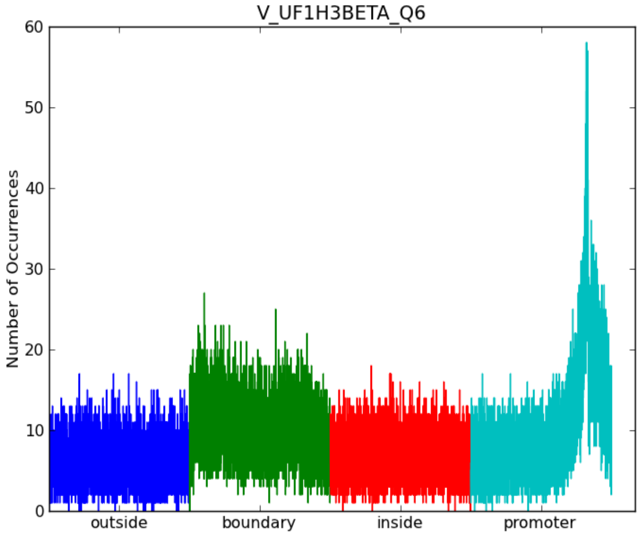

boundary: nan, promoter: nan

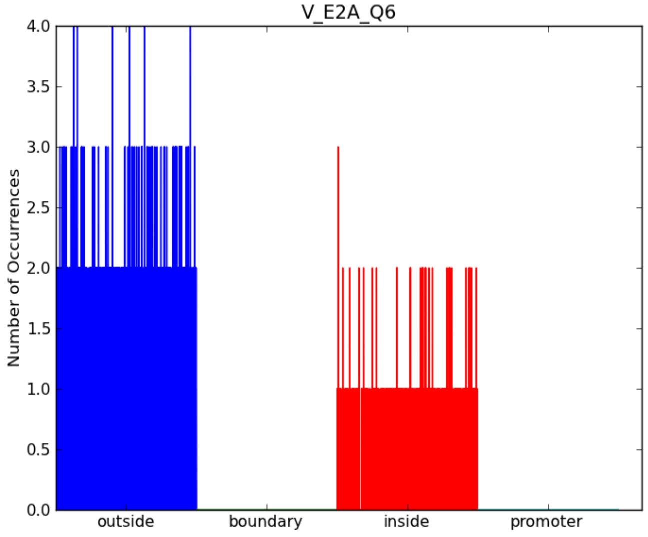

boundary: 2.34, promoter: 3.50

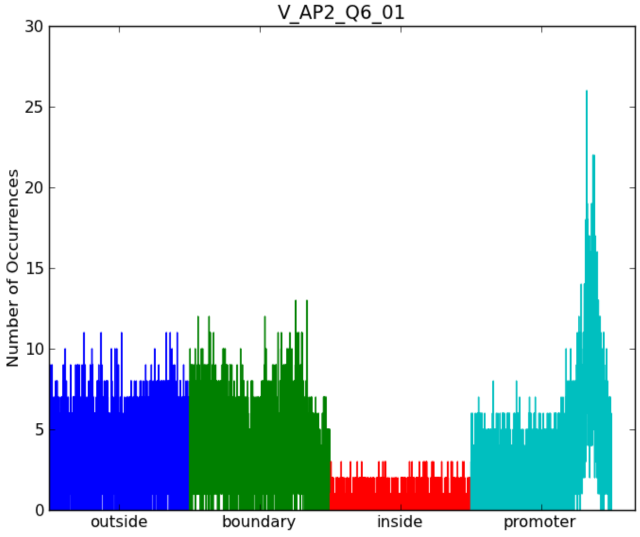

boundary: 4.84, promoter: 0.98

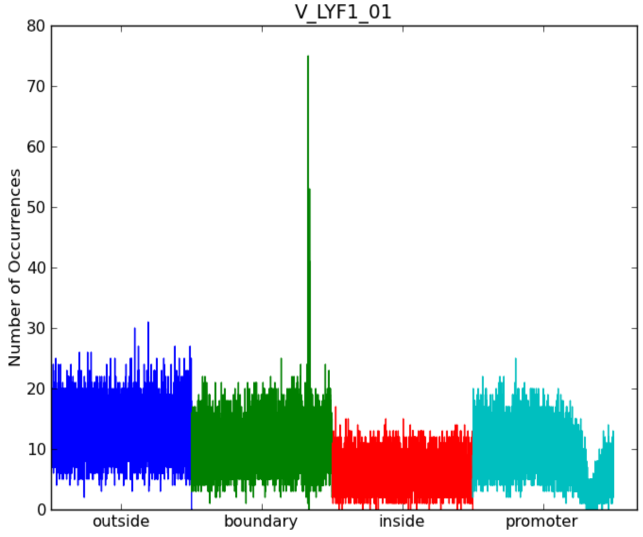

boundary: 3.23, promoter: 2.57

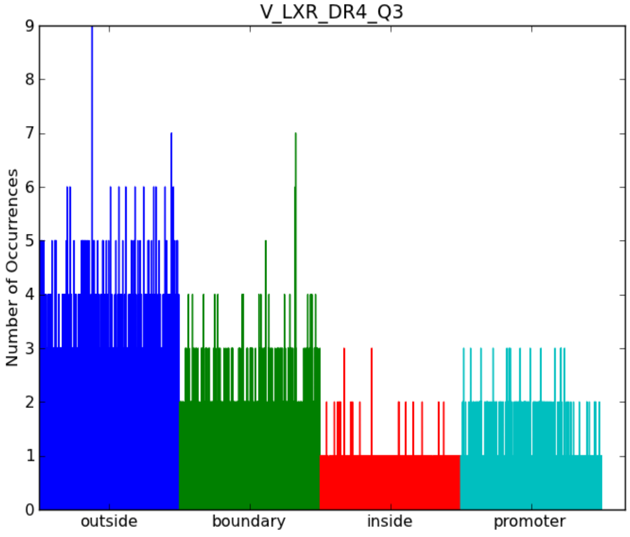

boundary: 3.12, promoter: 6.73

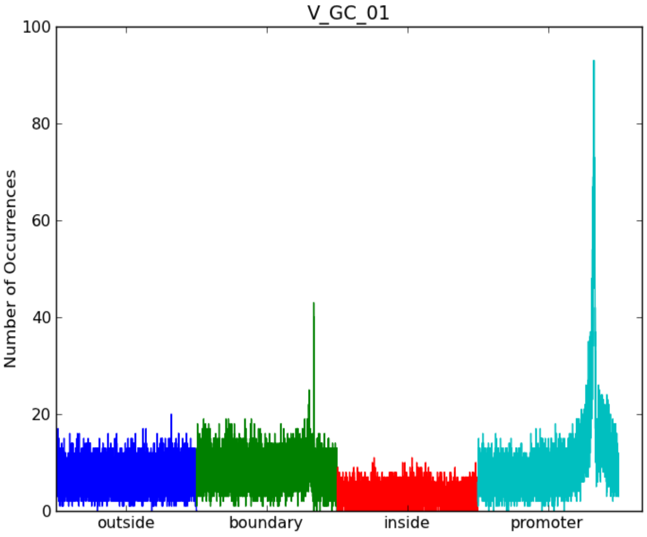

boundary: 3.38, promoter: 0.95

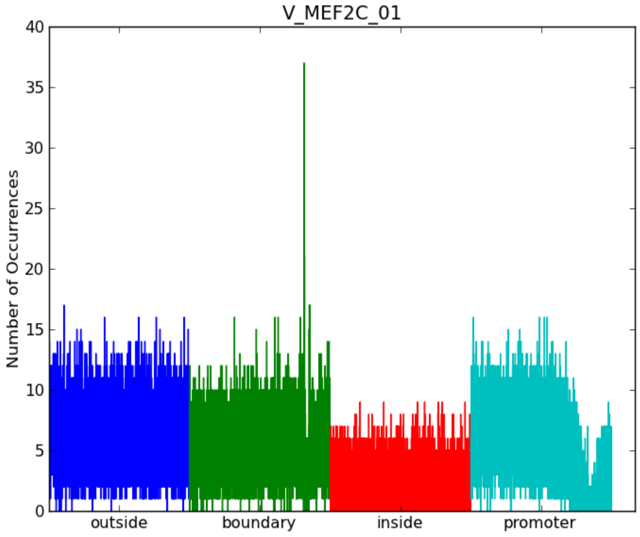

boundary: 3.35, promoter: 0.92

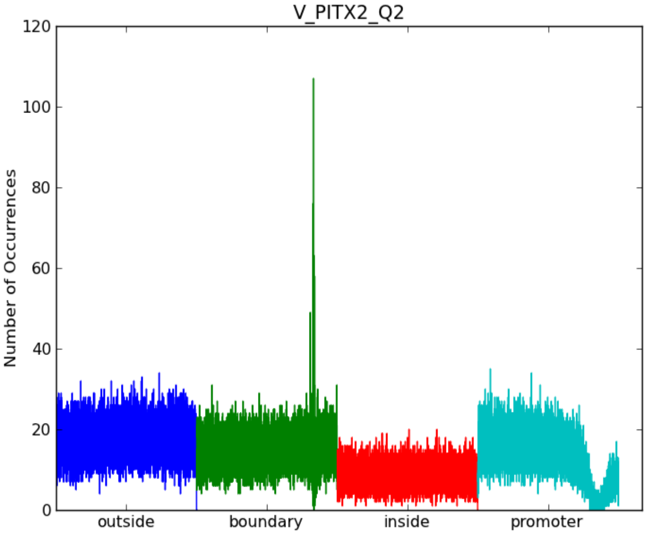

boundary: 4.35, promoter: 3.14

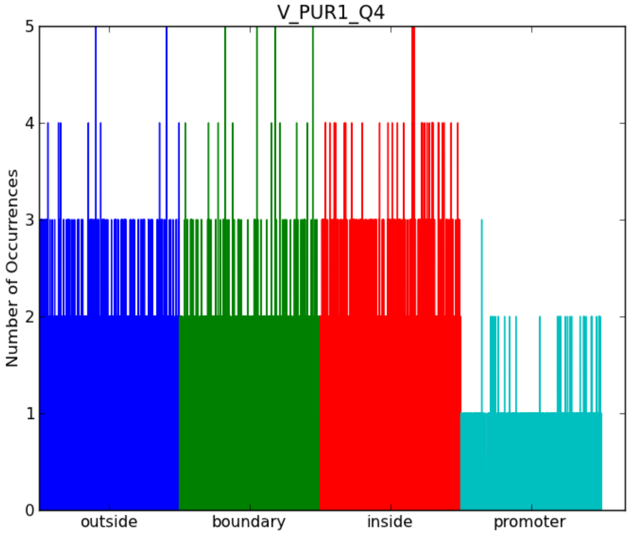

boundary: 3.48, promoter: 0.95

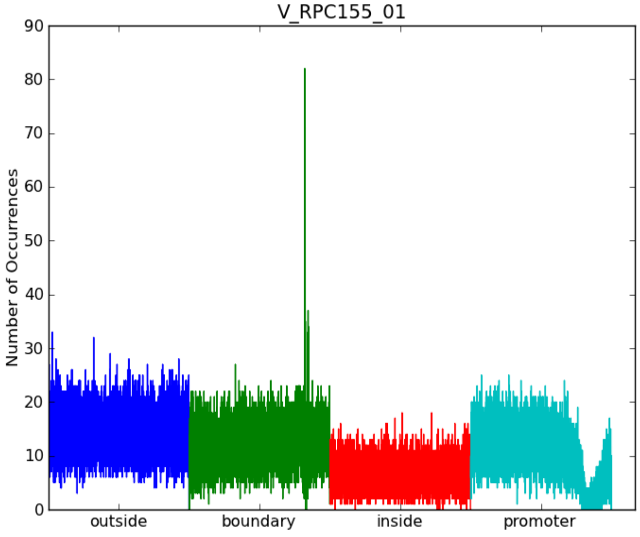

boundary: 4.06, promoter: 1.20

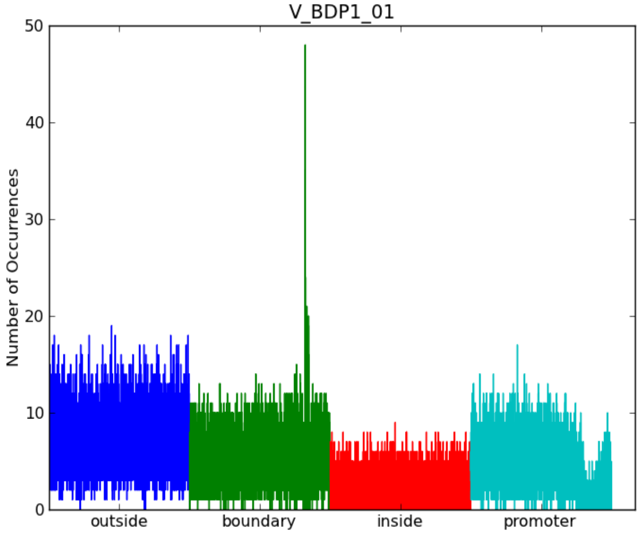

boundary: 2.97, promoter: 1.24

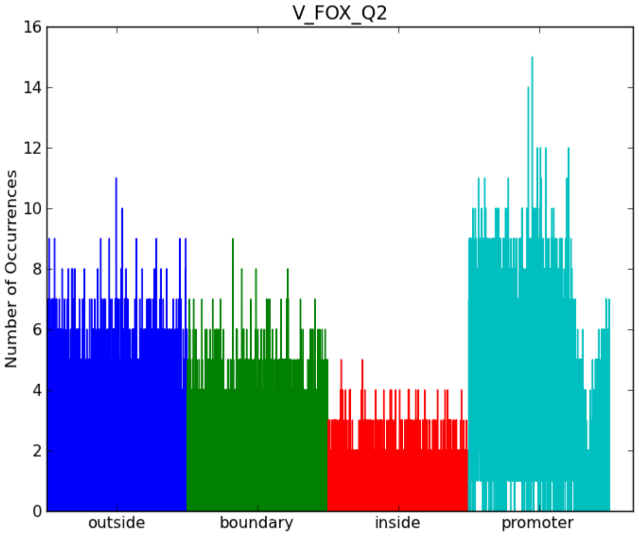

boundary: 4.67, promoter: 0.94

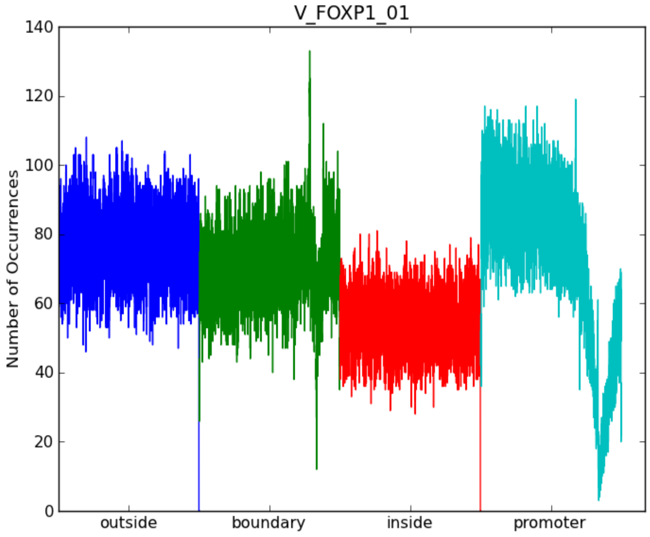

boundary: 3.39, promoter: 5.02

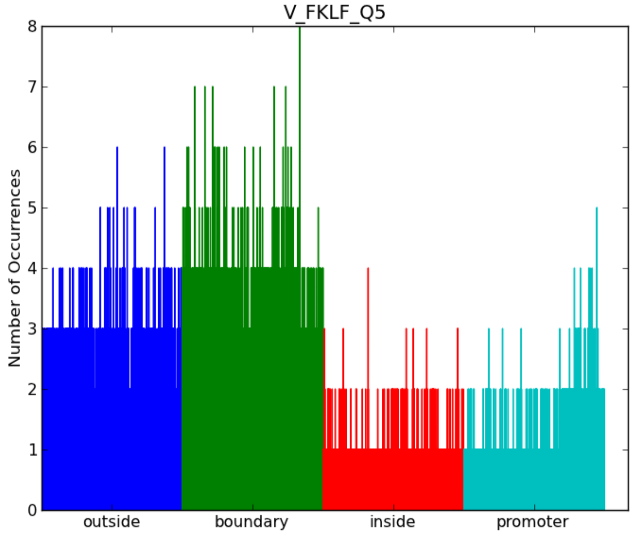

boundary: 2.98, promoter: 7.06

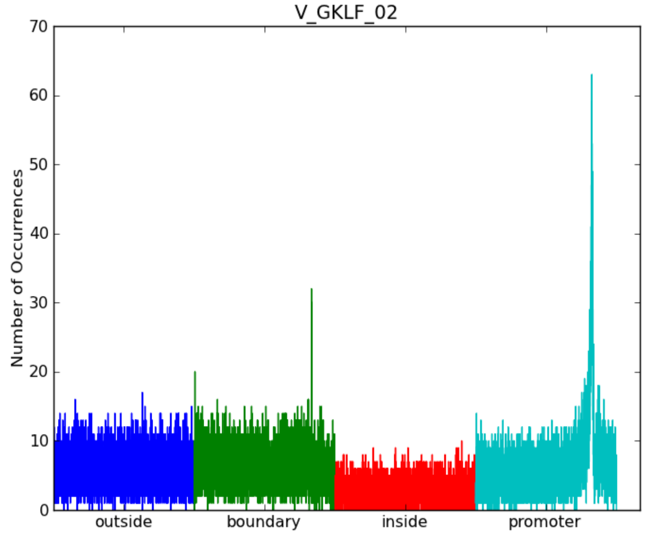

boundary: 2.45, promoter: 1.85

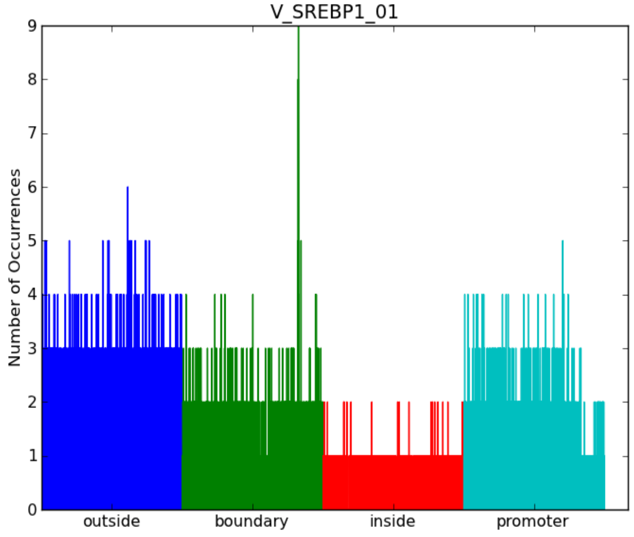

boundary: 1.92, promoter: 4.72

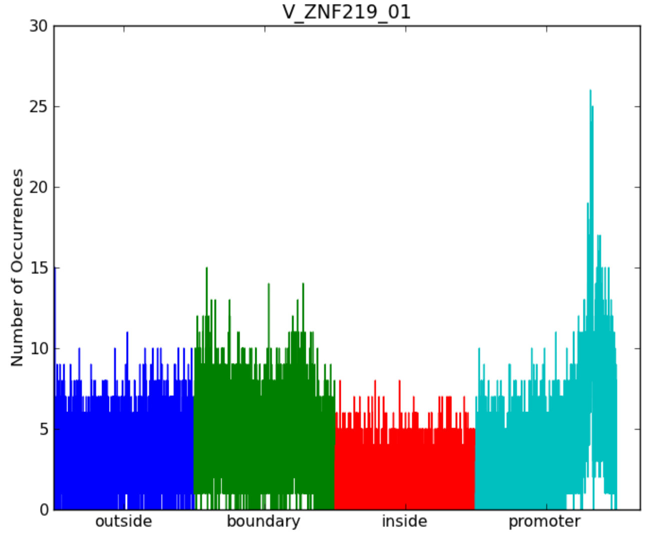

boundary: 4.31, promoter: 1.20

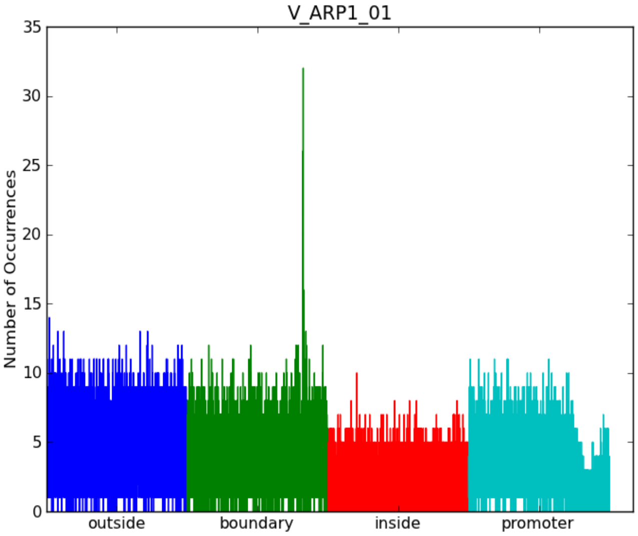

boundary: nan, promoter: nan

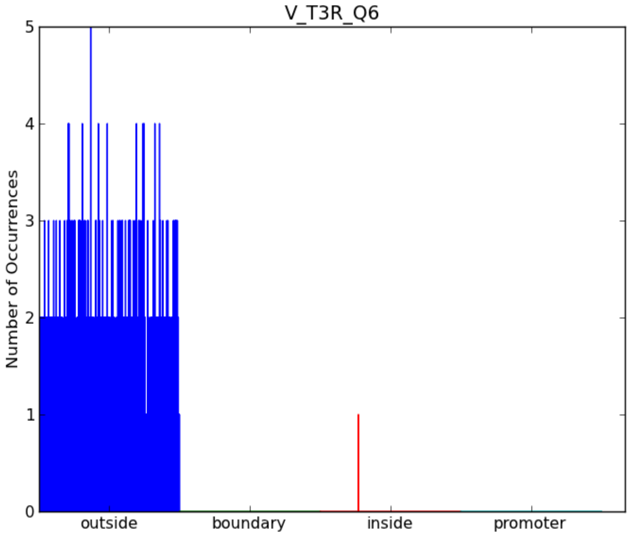

Supplement: Additional file 1: — Figure S1. Pattern of histone marks near HMB boundaries: (a) H3K4me1, (b) H3K9me3, (c) H3K27me3, (d) H3K36me3. Figure S2. ROC for HMB boundaries versus inside/outside for the test set. Figure S3. Frequency plots for the TF motifs listed in Additional file 2: Table S4. (PDF 1711 kb) [file 12885_2016_2128_MOESM1_ESM.pdf]
